# Injectable Hydrogel-Based Nanocomposites for Cardiovascular Diseases

**DOI:** 10.3389/fbioe.2020.00251

**Published:** 2020-03-31

**Authors:** Xiaoshan Liao, Xushan Yang, Hong Deng, Yuting Hao, Lianzhi Mao, Rongjun Zhang, Wenzhen Liao, Miaomiao Yuan

**Affiliations:** ^1^Department of Nutrition and Food Hygiene, Guangdong Provincial Key Laboratory of Tropical Disease Research, School of Public Health, Southern Medical University, Guangzhou, China; ^2^The Eighth Affiliated Hospital, Sun Yat-sen University, Shenzhen, China

**Keywords:** injectable hydrogel, nanocomposite, angiogenesis, stem cell homing, cardiovascular diseases

## Abstract

Cardiovascular diseases (CVDs), including a series of pathological disorders, severely affect millions of people all over the world. To address this issue, several potential therapies have been developed for treating CVDs, including injectable hydrogels as a minimally invasive method. However, the utilization of injectable hydrogel is a bit restricted recently owing to some limitations, such as transporting the therapeutic agent more accurately to the target site and prolonging their retention locally. This review focuses on the advances in injectable hydrogels for CVD, detailing the types of injectable hydrogels (natural or synthetic), especially that complexed with stem cells, cytokines, nano-chemical particles, exosomes, genetic material including DNA or RNA, etc. Moreover, we summarized the mainly prominent mechanism, based on which injectable hydrogel present excellent treating effect of cardiovascular repair. All in all, it is hopefully that injectable hydrogel-based nanocomposites would be a potential candidate through cardiac repair in CVDs treatment.

## Introduction

Cardiovascular diseases (CVDs), the group of pathological disorders, including atherosclerosis, myocardial infarction (AMI), stroke and heart failure (HF), remains the leading cause of death globally (Ujcic-Voortman et al., 2012; Nichols et al., 2014; Cainzos-Achirica et al., 2019). In the United States, there were 12.3 million deaths caused primarily by CVD from 2003 to 2017, among which, ischemic heart disease accounted for 48.2%, followed by cerebrovascular disease or stroke (16.7%), and heart failure or cardiomyopathy (10.6%) (Cross et al., 2019). CVDs affects the life of quality of patients, and causes enormous health and economic burdens (Gersh et al., 2010) all over the world, both in the developing countries (Lopez-Jaramillo, 2008; Gersh et al., 2010; Celermajer et al., 2012; McAloon et al., 2016) and in the rich ones (Gersh et al., 2010).

To date, current clinical regimens largely rely on the administration of drugs and other therapeutic agents such as the stem cell and the growth factors (Madonna and De Caterina, 2011; Bagno et al., 2018), basing on the hypothesis that a certain disease consists of dysfunctional cells and molecules within healthy organs and body. As well known, on the one hand, drugs or other therapeutic materials need to overcome physiological barriers to reach targets sites and during the process of transporting them, potential adverse effects may be produced; on the other hand, another hamper is the retention time of agents in the injury site is not adequate for new vessel growth. Therefore, the use of drug delivery systems (DDS) is necessary for enhancing the efficacy and safety of therapeutic agents (Matoba et al., 2017).

In the past decades, DDS has been investigated for improving the transportation efficiency of drugs or other agents of interest (Miyake et al., 1998). Currently, great advance about DDS has been made, for example, electrospun polymeric nanofibers (Torres-Martinez et al., 2018), Lipid-based DDSs (Semalty et al., 2009) and Metallic nanoparticles (Mody et al., 2010), Electrospun polymeric nanofibers (Torres-Martinez et al., 2018), as one of promising DDSs, has the capacity to improve drug’s bioavailability and release them in a controlled way via making the low solubility drugs loaded into the fibers. Besides, the high surface-to-volume ratio of the fibers can promote cell adhesion and proliferation, drug loading, and mass transfer processes. However, because of its high cost, the matter of manufacturing drug loaded electrospun mats has to be considered before wide utilization. As one of the lipid-based DDS, pharmacosomes (Semalty et al., 2009) were able to improve dissolution and absorption efficiency through the lipophilic membrane tissue owing to its amphiphilic property (Wang et al., 2011), so that the bioavailability of drugs was greatly improved. However, the targeting of the lipid-based DDS is still a challenge. Metallic nanoparticles (Mody et al., 2010) such as iron oxide nanoparticles have been widely used in targeted drug delivery since they were able to conjugate with antibodies and drugs of interest via modification of different chemical functional groups. However, the toxicity of these magnetic nanoparticles to certain kinds of neuronal cells remain unclear (Pisanic et al., 2007).

Recently, the utilization of injectable hydrogel-based DDSs has attracted considerable attention in many medicine fields, including chemotherapeutics (Norouzi et al., 2016), tissue engineering and regenerative medicine such as cartilage (Li J. et al., 2019) and spinal cord (Macaya and Spector, 2012). Injectable hydrogel has mechanical properties to closely match the targeting organ, and can also be loaded with cellular and a cellular therapeutics to modulate the wound environment and enhance regeneration (Frith et al., 2013; Seo et al., 2017; Cipriani et al., 2018; Mao et al., 2019). In the past years, hydrogels have been paid considerable attention as potential candidates for restoration of ischemia myocardial, in particular, those stem from natural extracellular matrix (ECM) components (e.g., collagen, fibronectin, as well as glycosaminoglycans) could favor greatly endothelial cells adhesion and their transformation to microvessels *in vitro* (Moon et al., 2010) attributing to their high water content and structural similarity to the natural ECM (Peppas et al., 2006; Seliktar, 2012). Additionally, when in an extremly swollen state, hydrogel-based materials such as chitosan hydrogels show good ability to deliver cells and bioactive agents (Liu et al., 2006). Besides, owing to its pH- and temperature-responsive properties, injectable hydrogel exhibits good capacities as a minimally invasive biomaterial scaffolding (Van Vlierberghe et al., 2011) applied for CVDs. Here, we review the wide application of various kinds of injectable hydrogel and the major strategies for the cardiovascular disease therapy.

## Single Use of Injectable Hydrogels

It is of significant potential for injectable hydrogels to be applied for cardiovascular diseases. The single use of injectable hydrogels characterized by minimally invasive has a suitable effect in cardiovascular disease treatment (Johnson and Christman, 2012). Injectable hydrogels are able to form a network structure at a certain temperature, to provide a morphological environment for supporting myocardial cells and retaining self-differentiated growth factors to promote myocardial repair (MacArthur et al., 2017). The current research and development focused on injectable hydrogels mainly divided into two categories: natural hydrogels and synthetic hydrogels.

### Natural Hydrogel

Natural hydrogels are attracting attention because of their non-toxicity, immunogenicity, and excretion of metabolites (Li L. et al., 2019). Generally, natural hydrogels are composed of polysaccharides or proteins whose water-swelling properties making them easy to adsorb and contain nutrients and small molecules (Ahmed, 2015) and improving cell survival and exercise performance (Ahearne, 2014).

Among them, the application of ECM (Extracellular matrix) hydrogel is the representative of natural hydrogel (Francis et al., 2017). Once the nanofiber hydrogel is formed by thermal induction at physiological temperature, the decellularized myocardial matrix hydrogels are possible to quickly create a natural cellular microenvironment for heart tissue and promote myocardial cell repair (Stoppel et al., 2016). Currently, ECM hydrogels are transformed into clinically available injectable biomaterial therapy stages by clinical trials (Wang and Christman, 2016). However, ECM is currently encountered with the lack of effective extraction methods with the reason that the use of chemical reagents for decellularization to remove the nucleus and cytokines of tissue organs can cause damage and denaturation of ECM proteins. Some scholars have proposed the use of supercritical carbon dioxide to extract to reduce damage while with an inevitable challenge of higher cost (Seo et al., 2018).

Therefore, there are many scholars who have developed other natural hydrogels and studied their role in promoting cardiovascular disease repair to replace ECM. Currently developed hydrogels biomaterials include chitosan natural hydrogels (Li J. et al., 2013), hyaluronic acid hydrogels (Yoon et al., 2009), sodium alginate hydrogels (Rocca et al., 2016), and so on. As an immunological linear neutral polysaccharide, hyaluronic acid has multiple acid and hydroxyl groups in the molecule, which can be modified into different forms of hydrogels, including soft or hard hydrogels, as well as nanoparticles and electrospinning. HA-based biomaterial (Burdick and Prestwich, 2011; Larraneta et al., 2018). The presence of reduced left ventricular volume of the glue, increased ejection fraction and the increased wall thickness evaluated by nuclear magnetic resonance (MRI) combined with finite element (FE) models following the treatment of injectable hyaluronic acid hydrogels confirmed the cardiovascular properties of injectable hyaluronic acid hydrogels, including mechanical properties and degradation properties which have been strongly verified before (Rodell et al., 2016).

Perivascular macrophages maintain the balance between endothelial cells and vascular permeability, but when exposed to foreign substances, they activate the inflammatory response and break the balance leading to vascular embolism (Lapenna et al., 2018). Fortunately, chitosan not only has a group that can be modified to change its properties (Vukajlovic et al., 2019), but also has good compatibility with macrophages (Aussel et al., 2019), suggesting that chitosan can treat cardiovascular diseases through vascular repair. Chitosan injectable hydrogels can also be used to remove free radicals due to their antioxidant properties and degradability, resulting in anti-inflammatory effects to promote heart and blood vessel repair (Dorsey et al., 2015). Similarly, due to the easy modification of chitosan, a suitable biocompatible conductive polypyrrole (PPy)-chitosan hydrogel was designed to effectively maintain myocardial function by connecting isolated cardiomyocytes to increase the electrical conductivity of cardiac tissue (Mihic et al., 2015).

In addition, the easily degradable, non-toxic sodium alginate hydrogel can be modified without modification and induced specific properties for a wide range of applications (Hadley and Silva, 2019). Once the degradable alginate hydrogel was designed to own a microstructure to sustain the release of angiopoietin, it can promote cardiac repair (Rocca et al., 2016) and that is why it widely used in cardiac engineering (Ruvinov and Cohen, 2016). According to the rapid development of alginate hydrogel, a multicenter prospective randomized controlled trial called AUGMENT-HF followed up for 1 year was conducted and found that patients with advanced heart failure (HF) using calcium alginate-injected hydrogel presented better cardiac function and clinical outcome rather than who accepting clinical standard medicine therapy (SMT) (Mann et al., 2016).

**FIGURE 1:**
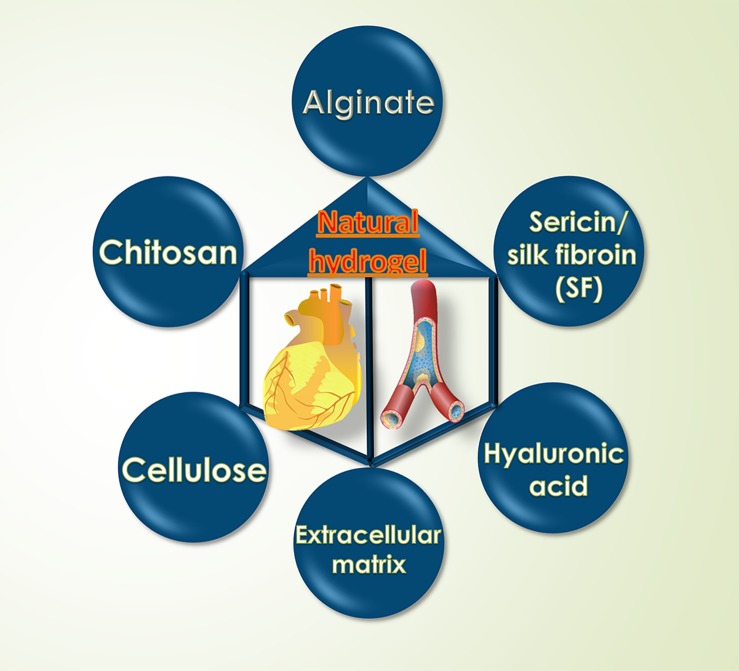
Common types of natural injectable hydrogels materials.

Furthermore, the sericin-injected hydrogel also performed an excellent biodegradability whose advantage is promoting the recovery of acute myocardial infarction (MI) by promoting inflammation and promoting cardiomyocytes and vascular repair, with limited application due to the high cost and weaker mechanical properties (Song et al., 2016). In order to improve mechanical properties, silk fibroin (SF) is used as a raw material for hydrogel to increase hydrogel toughness and obtain an appropriate degradation rate for better therapeutic effects (Kambe and Yamaoka, 2019). The following is a classification description of several common natural hydrogel materials (Figure 1).

Obviously, natural injectable hydrogels own good cardiovascular repair and biocompatibility, while the defects in uncontrolled function, rapid degradation rate, long gel formation time (Ahearne, 2014; Pena et al., 2018) and high production cost play (Song et al., 2016) a tough role of obstacle in the way to cardiovascular application. Therefore, the development of synthetic hydrogels had become researchers’ hot spot.

### Synthetic Hydrogels

Compared to natural hydrogels, synthetic hydrogels perform a strong mechanical properties and a possibility of being linked to new functional groups by physical and chemical means to achieve the desired function (Highley et al., 2016; Pena et al., 2018). Besides, extensively alternative synthetic materials range and the low risk of immune rejection implanted in the body (Wang R. M. et al., 2017) also facilitates the development of synthetic hydrogels. However, synthetic hydrogels are encountered with low adhesion, due to the lack of cell attachment sites, and poor biocompatibility (Do et al., 2015).

**FIGURE 2:**
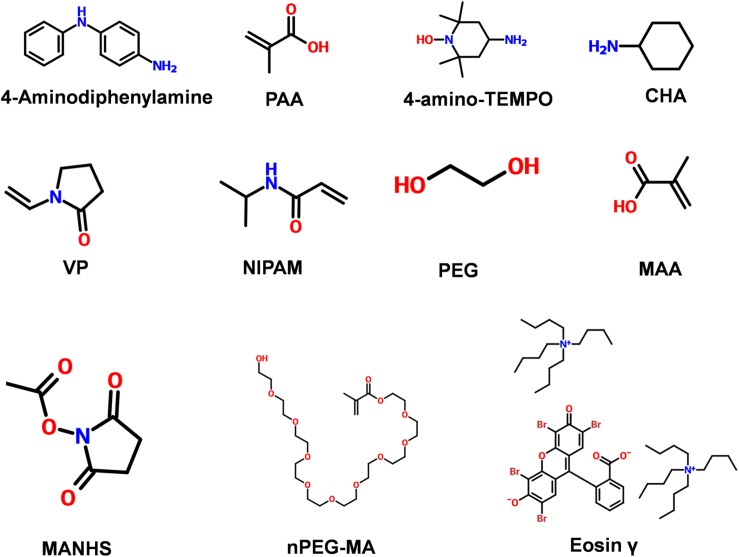
Commonly used chemical structure of synthetic materials. 4-Aminodiphenylamine; PAA (Poly acrylic acid); 4-amino-TEMPO; CHA (cyclohexylamine); VP (*N*-vinylpyrrolidone); NIPAM (*N*-Isopropyl acrylamide); PEG (poly ethylene glycol); MAA (Meth acrylic acid); MANHS (methacrylic acid *N*-hydroxysuccinimide ester); nPEG-MA [Poly ethylene glycol (n) monomethacrylate]; Eosin γ [Eosin γ bis(tetrabutylammonium salt) + 2-(2,4,5,7-tetrabromo-3-oxido-6-oxoxanthen-9 yl)benzoate,tetrabutylazanium].

The biochemical properties of hydrogels would be altered to be suitable to play a role in cardiovascular regeneration engineering due to the addition of chemical groups, the following is the introduction of several common synthetic hydrogels (Figure 2). The addition of 2-methylene-1,3-dioxepane (MDO) provided biodegradability, and the introduction of tetraaniline endowed copolymers with desirable electrical properties and antioxidant activities, were added to an *in situ* hydrogel composing of poly (NIPAM-based) copolymer that presented superior biocompatibility and conductivity (Cui et al., 2014). Furthermore, the biomimetic hydrogel visible-crosslinking with the GelMA provided biodegradability perform a good biocompatibility, while the biosafety of which has been questioned to some extent (Noshadi et al., 2017). It is worth noting that a functional polyion complex added by static cross-linking create a controlled release system of NO to inflammatory tissues to remove the ROS by redox reaction to promote angiogenesis and prolong the retention period for more than 10 days, which solved the problem of short retention time of natural hydrogel (Vong et al., 2018). Equally, the cross-linking with oxygen-suppressing microspheres to release oxygen to infarcted tissue increase myocardial cell survival rate (Fan et al., 2018). Clearly, the plasticity of synthetic hydrogels provides an effective way to treatment based on cardiovascular disease pathways. Moreover, the commercialization of synthetic hydrogels has developed rapidly owing to its designability. An injectable bioabsorbable stent (IK-5001) was used in patients with clinical MI before a 6-month follow-up, the result evaluated by laboratory examinations showed that IK-5001 was well tolerated without damage to the myocardium (Süselbeck, 2014). Undoubtedly, the prominent superiority of synthetic hydrogels are low manufacturing cost, low immunogenicity and controllability, which provides a huge space for the design and development, while biocompatibility, degradability and biosafety of synthetic hydrogels are issues worthily to be discussed.

Since injectable hydrogels have proven to be a good treatment in clinical practice (Wang H. et al., 2018) while the method is also encountered with the lack of suitable injectable hydrogel materials owing to the respective characteristics of natural hydrogels or synthetic hydrogels. Therefore, the exploration of clinically appropriate injectable hydrogel materials is also one of the research priorities (Table 1).

**TABLE 1:** Preclinical efficacy studies in the last 5 years using natural or synthetic injectable hydrogels for treating myocardial infarction.

Biomaterial	Type of hydrogel	Animal model	Type of MI model/Processing time point after successful MI model	End-point after treatment	Injection site	Results compared to control	References
Gelatinized alginate hydrogel	Natural	Rats	Acute myocardial infarction model/48 h	48 h/after 4 weeks	Myocardium	Associated with improved left ventricular function after MI in rats, and may provide a long-term supply of Angiotensin-(1-7)	Rocca et al., 2016
pcECM	Natural	Rats	Chronic myocardial infarction/12 weeks	4 weeks or 8 weeks	Myocardium	Preserved heart functions and alleviated MI damage	Efraim et al., 2017
Sericin	Natural	Mice	Acute myocardial infarction model/Immediate	6 weeks	Myocardium	Reduces scar formation and infarct size, increases wall thickness and neovascularization, and inhibits the MI-induced inflammatory responses and apoptosis	Song et al., 2016
hpECM	Natural	Rats	Acute myocardial infarction model/30 min	1 h	Myocardium	A significant reduction in scar volume along with normal electrical activity of the surviving tissue, as determined by optical mapping	Francis et al., 2017
Chitosan CSCl-RoY hydrogel	Synthetic	Rats	Acute myocardial infarction model	/	Myocardium	Improve angiogenesis at MI region and improve the cardiac functions	Shu et al., 2015
	Synthetic	Mice	Acute myocardial infarction model	4 weeks	Myocardium	Remarkably decreased the infarction size and improved the heart function	Vong et al., 2018
Type I collagen hydrogel	Synthetic	Rats	Acute myocardial infarction model/10 min	2 h, 1 and 28 days	Myocardium	Enhance the grafted cell survival in the myocardium, which contributed to the increased neovascularization, decreased interstitial fibrosis	Xia et al., 2015
TEMPO Gel	Synthetic	Rats	Acute myocardial infarction model/30 min	24 h	Myocardium	Reduced infarction/reperfusion injury and preserved left ventricle geometry	Zhu et al., 2018
HA	Synthetic	Ovine	Acute myocardial infarction model/30 min	8 weeks	Myocardium	Contractility in the BZ was significantly higher and ES fiber stress was also greatly reduced	Wang H. et al., 2018.

*MI, myocardial infarction; pcECM, porcine cardiac extracellular matrix cells; hpECM, human placenta-derived hydrogel; CSCL- Ro γ, chloride-Roγ hydrogel; TEMPO Gel, 2,2,6,6-tetramethylpiperidine-1-oxyl hydrogel; HA, hyaluronic acid; BZ, extension of the border zone; ES, end-systolic.*

## Injectable Hydrogel-Based Nanocomposites

**FIGURE 3:**
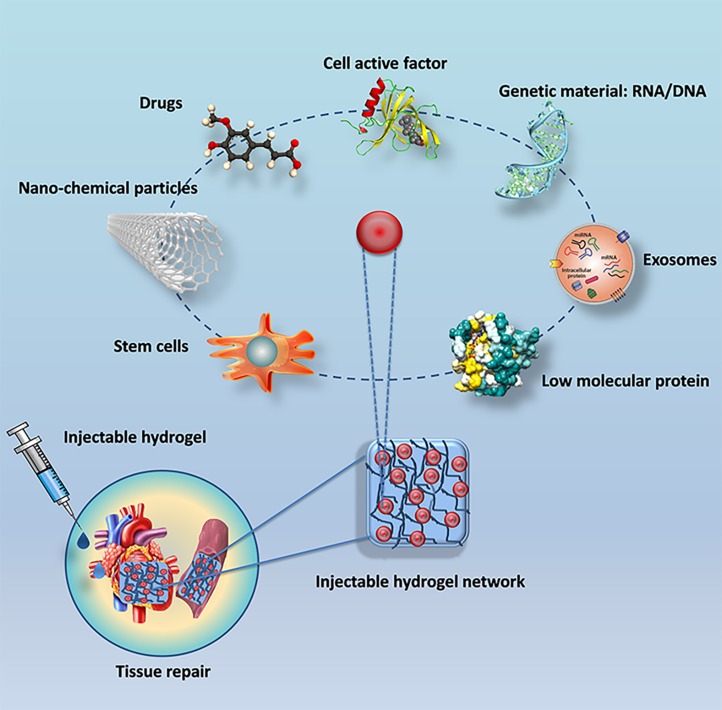
Common types of active nanomaterials complexed in injectable hydrogels for tissue repair.

It is urgent to develop an injectable hydrogel with a stronger intervention effect as the result of the suitable cardiovascular repair effect of a single natural or synthetic hydrogel usually dissatisfy the needs of clinical treatment. Generally, the hydrogels cross-linking with other substances present a better effect on the cardiovascular repair than which of hydrogel alone (Singelyn and Christman, 2011). It is significant that the nanofiber network structure of hydrogels provides a possibility of the combination with nanocomposites (Johnson et al., 2011). At the same time, in addition to the degradability of the injectable hydrogel (Tous et al., 2011), the particle size of the hydrogel (Yoon et al., 2014) is equally important to cardiovascular repair effects so that nanocomposite with hydrogel considered as a carrier plays a great potential role in the field of cardiovascular tissue engineering (Kurdi et al., 2010). We will review the common types of active nanomaterials complexed in injectable hydrogels for tissue repair as followed (Figure 3).

### Nanoparticles and Nanotubes

Limitations of clinical application of natural hydrogels and the lack of cell sites of synthetic hydrogel was ameliorated by the introduction of nanoparticles and nanotubes. Biocompatibility of nanoparticle composite injectable hydrogel have been demonstrated that the addition of nanoformulations into the ECM maintained the functional behaviors and balance of electrical conductivity into cardiomyocytes (Zhang et al., 2019). The addition of nanotubes not only avoided the low conductivity of natural injectable hydrogels, but also retained the strong mechanical properties of synthetic injectable hydrogels. A pHEMA [poly(2-hydroxyethyl methacrylate)] hydrogel consisting of RNT (rosette nanotubes) and CNF (carbon nanofibers) was designed to increase the conductivity of the myocardium and the mechanical properties to promote the adhesion of cardiomyocytes to enhance cells survival rate (Meng et al., 2013). In addition, the Au-loaded Laponite nanoparticles/ECM injectable hydrogel with superior electrical conductivity to reduces the long-short structure of the hydrogel to create a good environment for the cells (Zhang et al., 2019). Nanotube-injectable hydrogel can increase cell adhesion sites and ameliorate the arrangement structure of hydrogels to ensure cell-to-cell integrity to increase the survival rate of cardiomyocytes. For instance, carbon nanotube-incorporated collagen hydrogels can improve arrangement to promote cell–cell integrity and accelerate the regeneration of functional tissues in 3-D hydrogels (Sun et al., 2017). Recently, in addition to superior biocompatibility and electrical conductivity as well as appropriate adhesion sites, the nanotube composite injectable hydrogels were designed to provide sites for bioactive substance adhesion. In terms of vascular tissue engineering applications, Pacelli et al. (2017) proposes a Nanodiamond-based injectable hydrogel for controlled release of angiogenic factors since the chemical functional group on the surface of the ND efficiently interact with the VEGF and facilitate sustained release from the Polymer staggered network structure. The design of the bioactive substance adhesion site not only provides convenience for retaining the active factors produced by the cardiomyocytes itself, but also provides the possibility for carrying foreign biologically active factors. Significantly, the combination of bio-nanomaterials and tissue engineering is a definite effective means for cardiac tissue engineering.

### Drugs

Based on the insufficient therapeutic effect of oral medicine and cardiac stent treatment (Johnson and Christman, 2012), the drug-delivered injectable hydrogel treatment method, a minimally invasive surgical treatment, was proposed. Drugs or natural active substances can be introduced into the site of inflammation through using injectable hydrogels as carriers. Oxidative stress usually occurs with MI and lead to excessive generation of free radicals, which damages transplanted cell membrane lipid, proteins and DNA, seriously affecting the treatment of MI. Drug delivery with hydrogel can change the harsh environment of diseased tissue (Hasan et al., 2015). Hydrogels have a highly porous structure in which irregular pores are connected to each other throughout the structure (Trombino et al., 2019), and the drug or a biologically active substance like a liposome-encapsulated alpha-tocopherol (Qu et al., 2019) or Ferulic acid (FA) (Cheng et al., 2016) is uniformly distributed in the porous structure. The inlaid structure of the hydrogel creates a sustained release system to sustained-release to resists oxidative stress inflammatory response and improved cardiomyocyte survival rate. In addition to repairing blood vessels and promoting myocardial cell repair through the antioxidant action of biologically active substances, targeted therapy for drug delivery to damaged myocardium is also an effective means of treating adverse tissue remodeling. For instance, metalloproteinase inhibitor-containing injectable hydrogel was used to locally inhibit matrix metalloproteinases (MMPs), with the aim of reducing adverse tissue remodeling contributed by excess MMP activity (Purcell et al., 2014). At present, drug-encapsulated hydrogel treatment mainly focuses on finding suitable natural or chemical drugs that change the environment of tissue lesions, and designing suitable injectable hydrogel delivery systems. Moreover, the sustained-release effect of the DDS also affects the treatment of cardiovascular disease (Singh et al., 2019). It is worth noting that an injecting TIIA@PDA Nanoparticle-Cross-linked ROS-Sensitive Hydrogels as a nanoscale DDS roperly control of the drug release amount because TIIA@PDA NPs can be seized via the chemical bond between thiolate and quinone groups on PDA (Wang W. et al., 2019). There generally are a variety of sites of hydrogels that can be modified by reactive groups, such that the drug or active material to forms a composite gel by a cross-linking reaction such as a click chemistry or a supramolecular assembly of a guest-host pair (Highley et al., 2016). This design provides ideas for the development of sustained-release injectable hydrogels and it is an inevitable challenge of controlled release of the drug to be solved by the injectable hydrogel nanoscale DDS.

### Stem Cells

**FIGURE 4:**
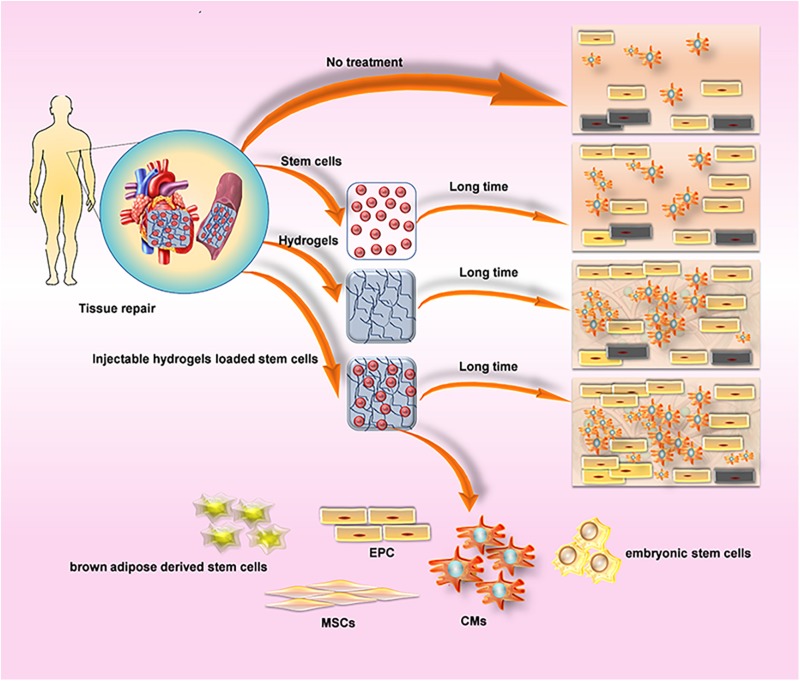
Comparison about the method of cardiovascular regeneration of hydrogel. **(A)** Cells Delivery produces paracrine effects, while hydrogels reduce the reduction of myocardial wall thickness, preserve heart function, prevent the formation of fibrous tissue, and provide a suitable environment for cell survival. Injectable hydrogels loaded cells suppress the reduction of wall thickness by Inhibiting physical tension to provide a suitable environment, and significantly improve the efficacy of cell therapy. **(B)** Commonly used cell types: CMs, cardiomyocytes; EPC, endothelial progenitor cells; brown adipose derived stem cells; embryonic stem cells; MSCs, mesenchymal stem cells.

Stem cell therapy, a treatment that has developed concurrently with drug-loaded injectable hydrogels therapy, is well known to play a very important role in cardiac engineering (Cheraghi et al., 2016). Hydrogels protect cells from host inflammation and enable functional integration with damaged myocardium by providing physical support for transplanted cells to maintain their location in the injured area (Sepantafar et al., 2016). Therefore, hydrogels for CVDs ought to be suitable for CMs owing to superior function in tissue repair (Figure 4). One of the aspects of current research on injectable hydrogels for transporting cells is to design a hydrogel that is more compatible with cells (Lovett et al., 2009). A polyethylene glycol (PEG) PEGylated fibrin proposed by Geuss et al. (2015) and an injectable hydrogel combained poly (propylene fumarate-co-sebacate-co-ethylene glycol) with PEGDA designed by Komeri and Muthu (2016) are also suitable for cardiomyocytes. In addition, hydrogel for CVDs should be electrically conductive to generate electrical signals to the myocardium (Sepantafar et al., 2016). An Injectable, flexible, antioxidant and electroconductive hydrogel with suitable biocompatibility, which is equivalent to CMs and provides a porous network structure suitable for embedding of CMs and sustained- generated electrical signal (Komeri and Muthu, 2017).

Myocardium contains approximately four basic cell types: 60–80% heart Fibroblast, 20–40% Cardiomyocyte (CM), smooth muscle cells (SMC) and endothelial cells (EC) (Dolnikov et al., 2006). It is necessary to recruit cardiac precursor cells to compensate for cell loss for high levels of cell slippage occurring during MI (Leri et al., 2005). Injectable hydrogel-based cell therapy techniques provide sufficient cell populations to support the ability to electromechanically couple to Cardiomyocytes (CMS) of host tissues, as well as provide appropriate vascular and connective tissue (Li and Weisel, 2014). The application and effects of various cell re-myocardia repair projects have been fully studied by researchers, among which embryonic stem cells (Lu et al., 2009) and CMs (Habib et al., 2011) are commonly used materials for cardiac engineering. In addition, mesenchymal stem cells (MSCs) are able to differentiate into cardiomyocytes for acute myocardial repair so that some researchers tried to combine the injectable hydrogel with MSCs to explore more effective therapeutic effects owing to the extremely low differentiation rate of MSCs in the heart and the function of hydrogel-injected network that provide a suitable environment and induce MSC differentiation (Li Z. et al., 2012). A tunable bioactive semi-interpenetrating polymer network (sIPN) hydrogels have been developed with matrix metalloproteinase (MMP) to create an assistive microenvironment for delivery of bone marrow-derived mesenchymal stem cells (BMSCs) into the Inflammatory myocardium. The cardiac function of the mice with the injection of hydrogel used as a carrier was improved which provided the basis for the long-term use of transplantation therapy for cardiac stem cells (Wall et al., 2010). Furthermore, an injectable hyaluronic acid (HA) shear-thinning hydrogel (STG) loaded endothelial progenitor cell (EPC) construct (STG-EPC) resulted in prolonged cell retention time and angiogenesis following injection into a myocardial infarction mouse model (Alarcin et al., 2018). In addition to the above-mentioned cells, researchers also used hydrogels to load human amniotic fluid stem cells (Yeh et al., 2010), cardiosphere-derived cells (Li Z. et al., 2011), brown adipose derived stem cells (Wang H. et al., 2014), autologous bone marrow cells (Chen et al., 2014) to promote cardiomyocyte differentiation and angiogenesis and so on (Table 2), and the achieved successful results among the cells above indicate that cell nanocomposites based on injectable hydrogels are a useful strategy for cardiac tissue engineering.

**TABLE 2:** Studies in the last 5 years using injectable hydrogels combined with cells for treating myocardial infarction.

Biomaterial	Type of cells	Animal model	Type of MI model/Processing time point after successful MI model	End-point after treatment	Injection site	Results compared to control	References
HA	Endothelial progenitor cell	Rats	Immediate acute myocardial infarction model	1/4 weeks	Myocardium	Minimize postischemic remodeling	Gaffey et al., 2015, 2019
FA	iPS	Mice	Immediate acute myocardial infarction model	1, 2, 4 weeks	Myocardium	Improved the retention and survival of iPS; less adverse heart remodeling and stimulation of neovascularization	Li H. et al., 2018
Chitosan	MSCs	Rats	1 weeks acute myocardial infarction model	24 h	Myocardium	Increased graft size and cell retention, promoted MSCs to differentiate into cardiomyocytes and increased the effects of MSCs on neovas-culature formation	Xu et al., 2017

*HA, hyaluronic acid; FA, folic acid; iPS, induced pluripotent stem cells; MSCs, mesenchymal stem cells.*

It is worth noting that the emerging 3D printing technology also provides a new idea for the design of injectable hydrogel cell nanocomposites (Table 3), since fine detail can be included on the micron level with high complexity which provide cells for a superior microenvironment with 3D printing (3DP) technology (Do et al., 2015; Kuo et al., 2015). There is no doubt that the cell composites based on the injectable hydrogel of 3D printing technology will be one of the hotspots of cardiac tissue engineering in the future (Alonzo et al., 2019; Han H. W. et al., 2019).

**TABLE 3:** Studies of using injectable hydrogels to formulate 3D structure for treating cardiovascular diseases.

Biomaterial	Formed 3D material	Loaded cells	Loaded time	Survival rate	References
Alginate	3D hydrogel-based vascular	L929 mouse fibroblasts	7 days	>90%	Gao et al., 2017
3D printable MEGEL/PEGDA3350/alginate hydrogel	3D hydrogel culture environment	HADMSC/HAVIC/HASSMC/	3 days	95%/93%/93%	Kang et al., 2017
Chitosan	3D hydrogel culture environment	NSCs/ECs	2 days	Survival rate in 3D hydrogel culture environment than that in 2D	Han H. W. et al., 2019
Plated PEGylated fibrin	3D hydrogel culture environment	HL-1 CMs	3 days	Increased cell retention and reduced scar tissue	Geuss et al., 2015

*3D printable MEGEL/PEGDA3350/alginate hydrogel, extrusion 3D printable mixture of methacrylated gelatin/poly-ethylene glycol diacrylate/alginate (MEGEL/PEGDA3350/alginate); PEG, Polyethylene glycol; HADMSC, human adipose derived mesenchymal stem cells; HAVIC, human aortic valve interstitial cells; HASSMC, human aortic valve sinus smooth muscle cells; NSCs, neural stem cells; ECs: endothelial cells; CMs, cardiomyocytes.*

### Cell Active Factor

The common injectable hydrogels-based treatments for cardiovascular disease are drug-loaded therapy and stem cell therapy, but both have limitations. The drugs currently in use are usually angiotensin receptor blockers, beta-blockers, angiotensin-converting enzyme (ACE) inhibitors and aldosterone antagonists, which possibly cause severe adverse reactions in patients, including sleep disturbances, hypotension and difficulty breathing (Jin and Yu, 2018). In order to clinically reduce the incidence of adverse reactions of CVDs, cell active factor therapy, including small molecule protein and exosomes, is considered as a cell-free treatment alternative to drug therapy (Cohen et al., 2014).

Endogenous and exogenous low molecular proteins are usually used in clinically applied cell-free therapies with difficult control of delivery and local release. Obviously, the application of injectable hydrogel probably significantly improved the biological activity of small molecule protein. An injectable hydrogel with a light-sensitive bond and photoresolvability, including polyethylene glycol and heparin-based polymers, successfully wrapped fibroblast growth factor 2 (FGF-2), whose activity was comparable to that before embedding and significantly altered the release profile of FGF-2 (Kharkar et al., 2017). It is worth noting that some researchers attempted to embed horseradish peroxidase (HRP) with a bioactive peptide with a phenolic hydroxyl group into hydrogel to cause a coupling reaction to enhance the function of the active peptide (Wang L. S. et al., 2014). The combination of injectable hydrogel and small molecule protein, which can not only improve its biological activity but also significantly increase the retention time of active protein in the myocardium and achieve sustained release, promotes cardiovascular repair by promoting cell homing and regulating key proteins. MacArthur et al. (2013) successfully loaded the synthetic analog of stromal cell-derived factor 1-α (engineered stromal cell-derived factor analog [ESA]) into an injectable hyaluronic acid hydrogel and successfully induced the persistence of endothelial progenitor cells Homing. Moreover, a delivery system of MMP-2 specific inhibitor peptide CTTHWGFTLC (CTT), which enables CCT to be released continuously within 4 weeks, effectively preventing ECM degradation worsens the condition (Fan et al., 2017). The fusion protein (TAT-HSP27), consisting of the heat shock protein 27 (HSP27) and transcriptional activator (TAT), loaded into microsphere/hydrogel combination delivery devices for controlled release behavior for prolonged periods because the heat shock proteins is a favorable target for protecting cardiomyocytes under environmental stimulation (Lee et al., 2009). Similarly, researchers designed low molecular protein injectable hydrogel nanocomposites with sustained release function according to the mechanism of action of different low molecular proteins: an Poly(ethylene glycol) dimethacrylate(PEGDMA) hydrogel storing local increasing mechano growth factor (MGF), a member of the IGF-1 family with an anti-apoptotic E domain playing a role of a stem cell homing factor (Doroudian et al., 2014), a temperature-sensitive chitosan chloride-RoY (CSCl-RoY) hydrogel (Shu et al., 2015), a hydrogel loading Neuregulin-1β (NRG) which is a member of the epidermal growth factor family (Cohen et al., 2014), a hydrogel loading high-mobility group box 1 (HMGB1) (He et al., 2013), and so on. Song M’s findings on association between stem cell homing factor (SDF-1) and angiogenic peptides (Ac-SDKP) also demonstrate a better therapeutic effect in combination with bioactive substances (Song et al., 2014).

In addition to the aforementioned small molecule regulatory proteins, certain growth factors, including Thymosin β4 (Tβ4), especially vascular endothelial growth factor (VEGF), should be delivered to heart tissue to reduced poor heart remodeling and improving ventricular function because of the poor cardiac remodeling that occurs later in the myocardial infarction (Anselmi et al., 2000). Thymosin β4 (Tβ4), a 43-amino acid peptide which performs angiogenic and cardioprotective properties, combined with injectable hydrogel resulted in stimulation of Vascular regeneration and cardiomyocyte migration (Shaghiera et al., 2018). Transportation of vascular endothelial growth factor (VEGF) and other angiogenic factors to promote angiogenesis are both potential treatment for cardiovascular disease and a vital aspect of tissue regeneration (Cao et al., 2009). The myocardial thickness and the density blood vessels of the rat myocardial infarction model were larger than that of the group without treating, following the injection of a novel temperature-susceptible aliphatic polyester hydrogel (HG) crosslinked with VEGF (Wu et al., 2011). Retention of highly vascularized cardiomyocytes is a limiting factor in growth factor therapy although it presents superior performance in cardiovascular repair (Rufaihah et al., 2017). The Dex-PCL-HEMA/PNIPAAm hydrogelcon containing VEGF developed by Zhu et al. (2016) and the injectable hydrogel amalgamated polyethylene glycol with fibrinogen (PEG-fibrinogen) loaded with VEGF-A designed by Rufaihah et al. (2013) both are able to release and store VEGF in a controlled manner and achieve better cardiac repair than VEGF alone. Moreover, in order to present a superior repair effect, an polyethylene glycol-fibrinogen (PF) hydrogels was manufactured for sustained dual transportation of VEGF and angiopoietin-1 (ANG-1) to promote myocardial therapy (Rufaihah et al., 2017). Recently, researchers’ research hotspots have shifted from the development of nano-growth factor injectable hydrogels to exploring which nano-growth factor injectable hydrogel complexes present superior cardiac repair functions. Therefore, the growth factors, including hepatocyte growth factor (HGF) (Ruvinov et al., 2010), insulin-like growth factor 1 (IGF-1) (Koudstaal et al., 2014; Fang et al., 2015), etc. that have been explored in combination with injectable hydrogels and present good myocardial repair effects, are suitable in myocardial regeneration.

Though stem cell treatment is one of the effective strategies for the CVDs, the stem cell clinical transplantation is limited by the low cell implantation and survival rate (Li Z. et al., 2018). Exosomes have recently become recognized as new candidates for cell-free treatment (Emanueli et al., 2015; Davidson et al., 2017; Zhang et al., 2017). Exosomes, extracellular vesicles derived from endosomes and the vital mediators of intercellular communication (Ibrahim and Marbán, 2016), are released by major cardiac cells, including cardiomyocytes, fibroblasts and endothelial cells (Barile et al., 2017), to regulate cellular function (Poe and Knowlton, 2018). Direct use of paracrine factors is an attractive strategy that play a role in therapy via cytokine regulatory pathway, taking cell implantation or survival rate out of considered (Han C. S. et al., 2019). The development of injectable hydrogel nanocomposites for composite exosomes has raise researchers’ attention since hydrogels are appropriate carrier materials. Significant improvement of exosome implantation on injured myocardium has been proven by that an injectable shear-thinning gel (STG) carrying EVs probably effectively improve myocardial function and increased the hemodynamics as well as the number of blood vessels (Chen et al., 2018). Furthermore, exosomes generated by human adipose-derived stem cells (hASCs), Gelatin and Laponite^®^ were combined to formulate a shear-thinning, nanocomposite hydrogel (nSi Gel) which was considered as an injectable carrier of secretome (nSi Gel+), and the results indicate an increasing density of blood vessels around the myocardium, an improvement in myocardial function and a reduction in scar area (Waters et al., 2018). However, the residence time and stability of exosomes are the major challenges in the clinical application of exosomes in recent years. Therefore, good biocompatibility and retention time are the vital research directions of exosomes-delivered injectable hydrogels. The stability and cardiovascular application of chitosan-injectable hydrogel-encapsulated paracrine factors *in vivo* were demonstrated by the results which indicated that exosomes showed high retention rates and promote vascular repair and formation (Zhang K. et al., 2018). Since the principle of stem cell therapy is based on the release of paracrine factors around the myocardial injury tissue to interfere with the progression of myocardial infarction (Mirotsou et al., 2011), exosome nanocomplexes with injectable hydrogels plays a significant role as promising alternative therapies.

### Genetic Material: RNA/DNA

Since stem cell and foreign active substance suppression is prone to collective immune rejection (Lu et al., 2010), embedding exogenous genetic material (DNA/RNA) into injectable hydrogels to produce autologous histocompatibility stem cells to promote myocardial regeneration, is an appreciated method in CVDs therapy. According to the pathway of MMP2 related to the cardiac harmful remodeling process, an injectable hydrogel complexed with siRNA up-regulate the hydrolytic activity of MMP2 protein to inhibit the harmful remodeling process of the heart and promote heart repair (Wang L. L. et al., 2018). In addition, an injectable Hyaluronan-Based hydrogel modulate remodeling of the myocardial extracellular matrix (ECM) by injecting a hyaluronic acid-based reservoir delivering exogenous microRNA-29B (miR-29B) (Monaghan et al., 2018). Noteworthily, protocols for injection-based delivery of Cre-CPP by ultrasound-guided injection to cardiac muscle in mice is mature owing to widely used technique of Cre-mediated DNA recombination at loxP sites (Chien et al., 2017), which provides a feasible mean for genetic material composite hydrogel. The study results above strongly demonstrated that genetic material (DNA/RNA) would be considered as the potential candidate for myocardial regeneration.

### Composite Use of Nano-Bioactive Substances

Cell therapy is currently the most mature treatment in cardiac tissue engineering which encounters with the problems of immune rejection of foreign cells, low survival rate and short residence time (Lu et al., 2010) so that researchers have begun to combine biologically active substance with stem cells to increase stem cell functional activity. It is common to carry out the mixture of cell growth factor and stem cells: combined polyethylene glycol hydrogel (PEG), a hydrogel consisting of human induced pluripotent stem cell-derived cardiomyocyte (iPSC-CM) and erythropoietin (EPO) (Chow et al., 2017), a hydrogel consisting of insulin-like growth factor (IGF-1) and delivering mesenchymal stromal cell (MSC) (Wang et al., 2010), injectable linear engineering protein hydrogels encapsulating VEGF and human induced pluripotent stem cell-derived endothelial cells (hiPSC-EC) (Mulyasasmita et al., 2014) and the like. What raise researchers’ attention is the combined use of multiple nano-bioactive substance. An injectable matrix metalloproteinase (MMP)-responsive, bioactive hydrogel used as an *in situ* forming scaffold to deliver thymosin β4 (Tβ4), along with vascular cells derived from human embryonic stem cells (hESC), which useful in engineering sustained tissue preservation (Kraehenbuehl et al., 2011). Noteworthily, Karam et al. (2014) proposed to integrate human adipose-derived stem cells (ADSCs) and pharmacologically active microcarriers (PAMs), a three-dimensional (3D) carrier of cells and growth factors, into an injectable hydrogel (HG), to obtain a system that stimulates the survival and/or differentiation of the grafted cells toward a cardiac phenotype. This study suggests that the use of 3D nanocomposites is one of the more effective means and a hot spot in cardiovascular repair development. From a gene therapy perspective, an injectable biocompatible hydrogel which can efficiently deliver a nanocomplex of graphene oxide (GO) and vascular endothelial growth factor-165 (VEGF) pro-angiogenic gene is significant for myocardial therapy (Paul et al., 2014), which suggested the feasibility of gene therapy combined with cardiac tissue engineering treatment is illustrate.

## The Major Mechanism Using by Injectable Hydrogel in CVDs

Although injectable hydrogel as a desirable candidate for CVDs with numerous outstanding properties has been widely used in clinical treatment, its mechanism of promotion restoration of CVDs remains unclear. Herein several possible paths are illustrated in the following parts.

### The Promotion Effect of Recovery in CVDs via Angiogenesis

**FIGURE 5:**
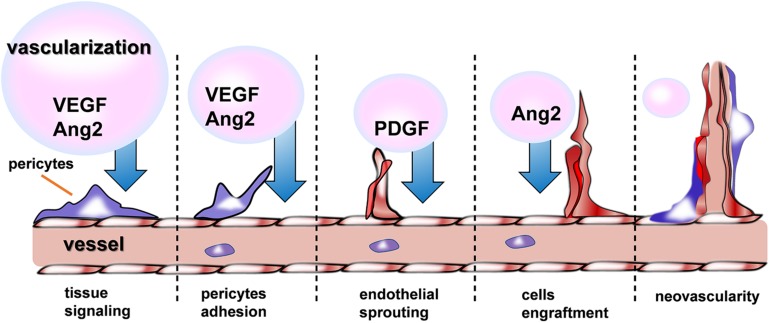
Schematic illustrations of different growth factors signaling during angiogenesis.

Recently, therapeutic angiogenesis, or the delivery of angiogenic agents such as growth factors (GFs) (Madonna and De Caterina, 2011), NO (Vong et al., 2018), and some drugs (Qi et al., 2018) to promote revascularization of ischemic tissue, holds great promise in the fields of treating CVDs. As shown in the Figure 5, a variety of GFs are indispensable for the different phrase of neovascularization. Nevertheless, this approach has been confronted with several obstacle when hydrogel used as a delivery device, among which, the difficulties of keeping angiogenic GFs retained locally at the injury site and released gradually to allow adequate time for growth of new blood vessels must be overcome before successful clinical implementation. Basing on the status, recently a growing body of evidences have shown evidence of injectable hydrogel’s promising effects on cardiac recovery through addressing the problems mentioned above in the process of revascularization.

GFs therapy shows great promises in treating ischemia, but the retention of GFs in the highly vascularized myocardium is mainly obstacle of its widely application. Some researchers (Feng et al., 2017) designed an injectable hydrogel scaffold composed of Konjac glucomannan (KGM, a naturally derived polysaccharide with capability to activate macrophages/monocytes to secrete pro-angiogenic/-mitogenic GFs) and heparin (Hep, one of the glycosaminoglycan molecule that binds numerous pro-angiogenic GFs and sequester them). Therefore, the injectable hydrogel was capable of promote revascularization via first stimulating the secretion of endogenous pro-angiogenic growth factors (GFs) and next sequestering these GFs inside the scaffold. Furthermore, controlling the degradation kinetics of injectable hydrogel would be an effective strategy to prolong the retention of GFs. Gel-CDH/HA-mCHO (Hozumi et al., 2018) hydrogels, a new injectable hydrogel synthetized by carbohydrazide -modified Gel (Gel-CDH) and mono-aldehyde modified-HA (HA-mCHO), was degraded much more slowly because of stable Schiff’s base formation between aldehyde and carbohydrazide groups. Additionally, the limited function of one single GF in the delivery system was one of the restrictions. Therefore, polyethylene glycol-fibrinogen (PF) hydrogels (Rufaihah et al., 2017) was employed and incorporating with vascular endothelial growth factor (VEGF) and angiopoietin-1 (ANG-1) to achieve the effect of dual delivery of GFs in a sustained release way. Besides, other materials also play the crucial roles on cardiovascular diseases. It is generally known the significance of nitric oxide (NO) but its therapeutic application is hampered because of its highly short half-life and rapidly consumed by excessive producing of ROS. Thereby, a new injectable hydrogel, namely NO-RIG (Vong et al., 2018), was prepared which consisted of PArg-PEG-PArg (NO releasing polymer) and PMNT-PEG-PMNT (ROS scavenging polymer), in a complex with polyanion PAAc, so that NO’s effect on promoting angiogenesis were improved.

In addition to the adequate retention time of GFs at the targeted district, transporting the GFs to the injury site accurately is also important for inducing angiogenesis. Given the acidic microenvironment (Khabbaz et al., 2001; Kumbhani et al., 2004; Ding et al., 2011; Zhao et al., 2012; Wei et al., 2017) of ischemic myocardium, a pH- and temperature-responsive, injectable hydrogel has been synthesized (Garbern et al., 2011) with several pH- and temperature-responsive random copolymer, including *N*-isopropylacrylamide (NIPAAm), propylacrylic acid (PAA), and butyl acrylate (BA) by reversible addition fragmentation chain transfer polymerization. This polymer existed as a liquid at room temperature and pH 7.4 but becomes a gel at 37°C and pH 6.8. Thereby, the hydrogel successfully provided sustained release of basic fibroblast growth factor (bFGF) at the injury site locally and the angiogenesis effect of bFGF were improved. Similar, another new (Wu et al., 2011), temperature-sensitive, aliphatic polyester hydrogel (HG) conjugated with (VEGF) was designed and also shown good therapeutic effect on attenuating adverse cardiac remodeling and improved ventricular function when injected after an MI.

In a word, therapeutic angiogenesis showed remarkably therapeutic potential in cardiovascular disorders by changing the status of one single material delivering, prolonging the retention of pro-angiogenic factor and transmitting them accurately to the targeted site.

### The Therapeutic Effect in CVDs Through Promoting Stem Cell Homing

**FIGURE 6:**
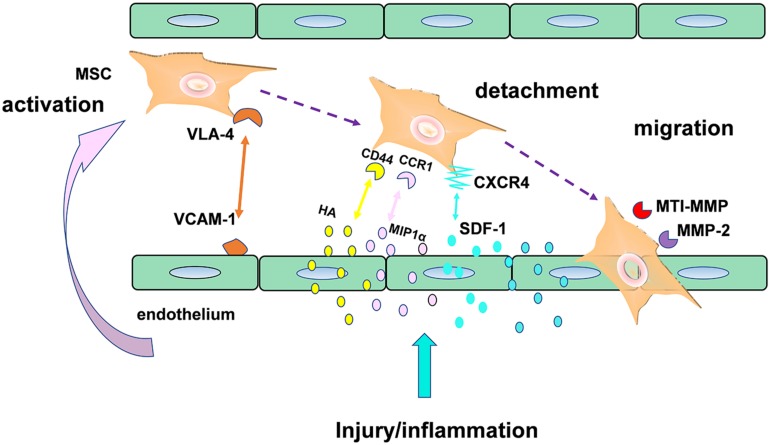
Mechanisms of MSC transendothelial migration toward injured tissue.

Stem cell homing, the capability of stem cells to find their destination in a targeted organ through the bloodstream (Zhao and Zhang, 2016), was another promising therapeutic strategy in CVDs, especially in Myocardial infarction (MI). Here, an example of mesenchymal stromal cells (MSC) in Figure 6 (Marquez-Curtis and Janowska-Wieczorek, 2013) was used to illustrate the mechanisms of stem cell transendothelial migration toward injured tissue. As we can see in the Figure 6, the effect of MSC homing was achieved by production of a series of some critical factors such as homing receptors including CXCR4. Although the mechanism of stem cell homing has been understood recently, the clinical utilization of stem cells was mainly hindered by their poor homing efficiency. In the recent years, a growing body of clinical evidence suggests that injectable hydrogel is a promising biomaterial that were capable of enhancing stem cell homing efficiency in treatment of numerous filed of regeneration medicine, such as in periodontal regeneration (He et al., 2019), cartilage regeneration (Lu et al., 2018), as well as corneal epithelium regeneration (Tang et al., 2017).

“Homing” directs stem cells migration through different signaling pathways, mediated by released chemokines or growth factor receptors on the surface of stem cells. Over the past decade, the most thoroughly studied stem cell homing factor is the chemokine SDF-1α/CXCL12 (Ghadge et al., 2011), based on which, a number of researchers committed themselves to develop some new delivery devices loaded with these promoting homing factors in order to improve the myocardium repair. Recently, a combined strategy (Naderi-Meshkin et al., 2016) was implemented via mixing human adipose tissue-derived MSCs (hASCs) into chitosan-glycerophosphate-hydroxyethyl cellulose (CH-GP-HEC) injectable hydrogel and as a result, site-directed homing efficacy and retention of ASCs increase by harnessing SDF1/CXCR4 axis. Similar, The E domain of mechano growth factor (MGF) (Doroudian et al., 2014) peptide is anti-apoptotic and a stem cell homing factor. As shown in a study, a microrod delivery device of poly (ethylene glycol) dimethacrylate (PEGDMA) hydrogel could absorb cells and decrease apoptosis of myocytes via incorporating MGF.

On the other hand, a comfortable microenvironment for stem cell survival is also of great significance. For example, as shown in a current report, ROS (Song et al., 2010) in MI microenvironment negatively regulated graft cell death and stem cell adhesion, finally caused anoikis of transplanted cells. Hence, changing the unfavorable MI microenvironment for stem cell homing and proliferation would have better therapeutic efficiency in cellular cardiomyoplasty. Chitosan hydrogel (Liu et al., 2012) were able to improve the MI microenvironment, enhance stem cell engraftment and survival through ROS scavenging. Furthermore, adequate blood vessel would be another crucial supportive condition for cell survival and proliferation. Thus, some scientists (Song et al., 2014) designed a biomimetic hydrogel incorporated with both stem cell homing factor (SDF-1) and angiogenic peptides (Ac-SDKP) in treating chronic myocardial infarction (CMI) and consequently, regeneration of cardiac function model were significantly promoted. By and large, the stem cell homing-based injectable hydrogel emerged as a promising therapy in treatment ischemic infarction.

All in all, as for treating CVDs, revascularization and stem cell homing are the two major effective strategies through injectable hydrogel as a delivery system in the recent years. Besides, there existing other approach that would hold great therapeutic potential in the field of CVDs treatment, for instance, taking advantage of an injectable hyaluronic acid (Zhang Y. et al., 2018) (HA) hydrogel to deliver miRNA in order to induce proliferation in cardiomyocytes through its inhibition of Hippo signaling via a direct binding site on the 3′ UTR, such as miR-302 (Wang L. L. et al., 2017) and miR-1825 (Pandey et al., 2017), developing a new hydrogel (Qi et al., 2018) from supramolecular assembling of a synthetic glycol peptide which endows the hydrogel with the capacity of endothelial cell adhesion and proliferation due to its high density of glucose moieties, as well as using Ferulic Acid (Kanki and Klionsky, 2009; Wu et al., 2012; Wiley et al., 2013) (a natural antioxidant that is most abundant in vegetables, especially in eggplants and maize bran) to form a new injectable hydrogel (Cheng et al., 2016) to effectively promote the recovery of Cisd2 deficiency induced damage.

## Summary and Perspective

Injectable hydrogels have shown promise in promoting cardiovascular disease repair for years from single hydrogels (natural or synthetic hydrogels) to hydrogel- based nanocomposite. To the begin, natural hydrogels were attracting attention because of their non-toxicity, immunogenicity, and excretion of metabolites (Li L. et al., 2019), such as. However, due to the lack of effective extraction methods (Francis et al., 2017), ECM were gradually replaced by other natural hydrogels, such as hyaluronic acid hydrogels (Yoon et al., 2009) (an immunological linear neutral polysaccharide with multiple acid and hydroxyl groups, which can be modified into different forms of hydrogels, including soft or hard hydrogels, as well as nanoparticles and electrospinning), chitosan natural hydrogels (which had good compatibility with macrophages and antioxidant properties and degradability) (Aussel et al., 2019), sodium alginate hydrogels (Hadley and Silva, 2019), and so on. On the other hand, synthetic hydrogels have been attached much importance since their strong mechanical properties and various and controllable function by physical and chemical means (Pena et al., 2018). Synthetic hydrogels are low manufacturing cost and could provide a huge space for the design and development, while their biocompatibility, degradability, biosafety and low adhesion for cell (Do et al., 2015) are issues worthily to be discussed.

Recently, since the porosity of hydrogel of hydrogels provides a possibility to combine with nanocomposites (Johnson et al., 2011), and the hydrogels cross-linking with other substances show better cardiovascular repair effect than which of hydrogel alone (Singelyn and Christman, 2011), several types of active nanomaterials complexed in injectable hydrogels for tissue repair have been explored. For instance, injectable hydrogel-based composite carrying drug and/or other bioactive materials have been explored and the effective have been achieved.

According to different treatment mechanisms and different aspects of concern, the invention of different nano-composite injectable hydrogels was designed. For example, drugs-delivered injectable hydrogels mainly improve the environment of myocardial tissue with excessive oxidative stress, and small molecule proteins-delivered and exosomes-delivered injectable hydrogels are mainly involved in the mechanism of hormone regulation in the process of self-repair of myocardium. Cell-delivered injectable hydrogels therapy is mainly to provide a large number of favorable healthy cells to promote the process of myocardial repair, while pure hydrogel therapy is mainly to provide the stent of myocardial cells. Recently, because of foreign material is prone to collective immune rejection, embedding foreign genetic material (DNA/RNA) into injectable hydrogels might be an appreciated method in CVDs therapy.

Although much progress has been made due to injectable hydrogel’s wide application in the CVDs, some limitations remain challenges that need to be overcome before successful clinical implementation, for instance, the exploration of appropriate approach for injection (Chen et al., 2017), the method for controlling and tailoring release profiles of targeting agents confronting the complicated biological processes (Kharkar et al., 2013; Annabi et al., 2014; Yesilyurt et al., 2016), the substantial requirement for hydrogel’s rheological and mechanical properties (Unterman et al., 2017), their capacities to be scaled up to a good manufacturing practice (cGMP) process (Ungerleider and Christman, 2014). It is of great hope that advances will be made along with our thorough study of the pathophysiology of CVDs and the accurate therapeutic mechanism by hydrogel in the treatment of CVDs in coming years. What is currently lacking is the comparison of the effects of different injectable hydrogels and that of their respective advantages in clinical applications. Although there are various designs of nanocomposite injectable hydrogels, their cost and clinical application are in controversy. Discussion and application of this composite product at this stage is Insufficient. In the future, we will focus on the rationality of the research design in this area and the possibility of clinical application.

## Author Contributions

All authors listed have made a substantial, direct and intellectual contribution to the work, and approved it for publication.

## Conflict of Interest

The authors declare that the research was conducted in the absence of any commercial or financial relationships that could be construed as a potential conflict of interest.
